# Variability, Expression, and Methylation of *IL-6* and *IL-8* Genes in Bladder Cancer Pathophysiology

**DOI:** 10.3390/ijms24076266

**Published:** 2023-03-27

**Authors:** Radosław Grębowski, Joanna Saluk, Michał Bijak, Janusz Szemraj, Paulina Wigner

**Affiliations:** 1 Department of Medical Biochemistry, Medical University of Lodz, 90-001 Lodz, Poland; 2 Department of Urology, Provincial Integrated Hospital in Plock, 09-400 Plock, Poland; 3 Department of General Biochemistry, Faculty of Biology and Environmental Protection, University of Lodz, 90-236 Lodz, Poland; 4 Biohazard Prevention Centre, Faculty of Biology and Environmental Protection, University of Lodz, 90-236 Lodz, Poland

**Keywords:** bladder cancer, single nucleotide polymorphisms, mRNA expression, methylation status

## Abstract

Bladder cancer (BC) is the 10th most common form of cancer globally, but its complete aetiology is still unknown. Nevertheless, there is evidence that chronic inflammation plays a role in the development and progression of BC. Therefore, the presented study aimed to detect a potential association between selected single nucleotide polymorphisms (SNPs)—rs1800797 and rs2069845 in *IL-6* and rs2227307 in *IL-8*—and BC development, as well as to identify the impact of BC on the level of expression and methylation of *IL-6* and *IL-8* promoters in PBMCs with the use of the TaqMan SNP genotyping assay, TaqMan gene expression assay, and methylation-sensitive high-resolution melting techniques. We did not find any association between the genotypes and combined genotypes of all studied polymorphisms and the occurrence of BC. However, we found that BC patients were characterised by decreased *IL-6* and *IL-8* mRNA expression levels compared to the controls. Additionally, the methylation status of the *IL-6* promoter was higher in controls than in BC patients. Our findings suggest that inflammation may be involved in the development and progression of BC.

## 1. Introduction

According to World Health Organisation (WHO) data, in 2015, cancer was the most common cause of death before the age of 70, thus constituting the most crucial barrier to extending life expectancy in every country in the world in the 21st century. Bladder cancer (BC) is the 10th most common form of cancer globally, with an estimated 573,000 new cases and 212,536 deaths in 2020. In addition, BC affects men much more often than women, with morbidity rates of 9.5 and 2.4 per 100,000 people, respectively, making BC approximately four times more common in men than women worldwide [1,2]. Thus, BC, the most common cancer of the urinary system, is diagnosed in 75% of men in the world’s population [3]. The highest incidence of BC is in Southern Europe (including Greece, with the highest male incidence rate in the world, Spain, and Italy), Western Europe (Belgium and the Netherlands), and North America. Interestingly, the highest rate of the disease among women was recorded in Lebanon [1,2]. Moreover, gender, age ≥ 75 years, smoking, low daily fluid intake, and industrial chemicals, including aromatic amines, are risk factors for BC [4,5,6,7,8,9,10]. In addition to numerous environmental factors that increase the risk of developing BC, the results so far also indicate the role of genetic factors in the pathophysiology of this disease. Interestingly, first-degree relatives of patients with BC had a two-fold increased risk of developing this cancer [11,12]. However, a recent genome-wide association study (GWAS) adjusted for occupational and environmental factors for bladder cancer susceptibility confirmed that the influence of genetic factors favouring the development of BC was modified by environmental factors, especially stress stimulation and work environment [13].

The transition of normal urothelium into bladder carcinoma is a multifactorial process. Thus, despite numerous studies, the mechanism of BC development remains unclear. Previous studies showed that chronic inflammation contributes to the initiation and progression of the underlying pathophysiology of invasive and metastatic cancer [14]. These findings formed the basis of the theory of immunoediting. This theory implies that while the human immune system protects from cancer, it also drives the development of tumours that will undergo immunogenic “sculpting” and which may survive immune cell attacks [15]. At the same time, other research results underlined the crucial role of oxidative stress in carcinogenesis, including the development of BC. The overproduction of free radicals is associated with damage to nucleic acids [16]. Moreover, reactive oxygen species (ROS) affect the activity of crucial biochemical pathways, including triggering inflammation by activating excessive production of pro-inflammatory cytokines such as IL-6 (interleukin 6) or IL-8 (interleukin 8) [17]. In turn, cytokines including IL-6 and IL-8 and their receptors may mediate the malignant transformation of urothelial cells and BC progression by appropriate transcription factor activation [18,19]. Therefore, the appearance of any mutations or SNPs in interleukins and their receptor genes may lead to significant changes in their biology and function and may be associated with a wide range of diseases and disorders, including cancers [20,21]. Previous studies confirmed that SNPs localise in genes linked with the inflammation, including genes encoding the Toll-like receptor (TLR), interleukin 1 (*IL-1*), *IL-6*, and *IL-8* [20,21,22,23]. In addition to polymorphisms and mutations, RNA-Binding Proteins (RBPs) and microRNAs (miRNAs) play a crucial role in the regulation of gene and protein expression by influencing assembly, modification, transport, localisation, stabilisation, degradation, and translation of RNA [24]. A previous study showed that RBM3 (one of the RBPs) might affect the mRNA stability of *IL-8* translation by binding to the 60S ribosomal subunit in an RNA-independent manner, increasing the formation of active polyribosomes, dephosphorylating eukaryotic initiation factor (eIF2α), and promoting eIF4E phosphorylation. In turn, *IL-6* expression is regulated by the RNA-binding protein LIN28, which inhibits let-7 maturation by blocking the inhibitory effect of let-7 on *IL-6* expression, which, in turn, may activate NF-κB (nuclear factor kappa-light-chain-enhancer of activated B cells) expression. Ultimately, abnormal activation of NF-κB leads to tumour development and may also be the cause of increased tumour aggressiveness and poorer prognosis [24].

In light of data indicating the crucial importance of IL-6 and IL-8 in carcinogenesis, our study aimed to determine the role of *IL-6* and *IL-8* genes in the molecular mechanism of BC development by evaluating the potential association between three polymorphisms located in genes encoding interleukin (-597 A>G (rs1800797) and c.3331 G>A (rs2069845) in *IL-6*, c.+396 T>G (rs2227307) in *IL-8*) and the development of BC in a Polish population. Moreover, we verified the impact of BC development on the mRNA expression and the methylation status of the promoter regions of *IL-6* and *IL-8* in the blood (PBMCs—peripheral blood mononuclear cells).

## 2. Results

### 2.1. Characteristics of Study Participants

Table 1 presents demographic and epidemiological factors for BC for the study patients and controls. The mean ± SD age for patients with BC was 69.67 ± 11.26 (range: 20–92), and the mean ± SD age for controls was 66.71 ± 11.76 (range: 28–91). Our analysis confirmed a significant difference between the distribution of marital status (single, married, and widow/widower), professional activity (physical work, mental work, unemployment, and pension), and co-occurrence of comorbidities, including hypertension and hypercholesterolaemia (yes vs. no) among patients and controls (*p* < 0.05). Moreover, significant differences were also observed for haematological and biochemical blood parameters. BC patients were characterised by lower levels of RBC, HCT, HGB, WBC, and potassium than controls (*p* < 0.05), whereas RDW, glucose, and creatinine concentrations were higher in the BC group (*p* < 0.05). Dipstick urinalysis reflected that urine samples of patients with BC showed protein (*p* < 0.001) and bilirubin (*p* < 0.01) presence compared to the absence observed in controls. Moreover, the urine of BC patients was cloudier than control urine (*p* < 0.001). Interestingly, urine microscopy analysis confirmed that the urine of patients with BC contained more RBC (*p* < 0.001), WBC (*p* < 0.01), and bacteria (*p* < 0.001) in the field of view.

### 2.2. Single Nucleotide Polymorphisms of IL-6 and IL-8 as the Risk of BC Occurrence

The genotype and allele distributions of the -597 A>G and c.3331 G>A polymorphisms of the *IL-6* gene in patients with BC and controls are presented in Table 2. The observed genotype frequencies were calculated in agreement with the Hardy–Weinberg equilibrium for controls and patients (*p* > 0.05, data not shown). The difference in the frequency distributions of genotypes of the polymorphism between the cases and controls was not statistically significant (*p* > 0.05). We did not find any correlation between genotypes/alleles of these polymorphisms and BC occurrence. Similarly, in the case of the c.+396 T>G polymorphism of *IL-8*, we did not find any correlation (*p* > 0.05) between genotypes/alleles of this polymorphism and BC occurrence (Table 2).

### 2.3. Association between Combined Genotypes of IL-6 and IL-8 Polymorphisms and BC Risk—Gene–Gene Interaction

We also analysed the association between the occurrence of BC and combined genotypes of studied polymorphisms, and these results are presented in Supplementary Table S1. Unfortunately, we did not find any correlation between the combined genotypes of all polymorphisms and the development of BC. Moreover, we performed SF analysis (Supplementary Table S2) according to the recommendations of Mario Cortina-Borja et al. (2009) [25], but we did not find any interactions between studied polymorphisms.

### 2.4. Linkage Disequilibrium and Haplotype Analysis

**Figure 1:**
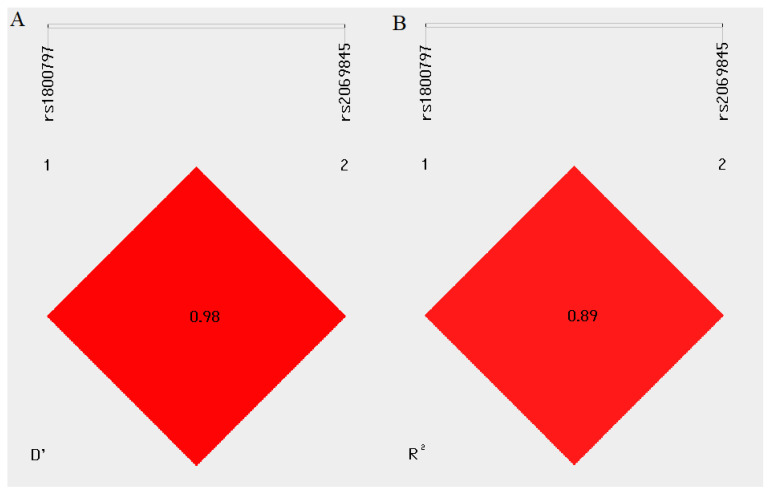
Analysis of linkage disequilibrium of the rs2069845 and rs1800797 polymorphisms in the *IL-6* gene. Pairwise D’ values (**A**). Pairwise R^2^ values (**B**). R^2^ ≥ 0.8—high LD.

Through LD analysis of the studied SNPs in the *IL-6* gene, we identified the rs2069845 and rs1800797 polymorphisms as severe linkage disequilibrium regions in *IL-6* (R^2^ ≥ 0.8) (Figure 1). Haplotype distribution of the 597 A>G and c.3331 G>A polymorphisms of *IL-6* is shown in Supplementary Table S3. Unfortunately, we did not observe a significant link between the haplotypes of the two polymorphisms and the occurrence of BC.

### 2.5. The Association between Studied Polymorphisms and the Clinical Histopathological Parameters of BC Patients

We assessed the association between the studied polymorphisms and primary tumour size, the status of lymph node metastasis, and distant metastasis (Supplementary Table S4). For this, we divided BC patients into subgroups according to the size of the primary tumour (Ta, T1, ≥T2), the status of lymph nodes (N0, ≥N1), and distant metastasis (M0, M1) according to the 8th edition of the TNM Classification of Malignant Tumours developed by the Union for International Cancer Control (UICC) and used by the American Joint Committee on Cancer (AJCC) and the International Federation of Gynaecology and Obstetrics (FIGO) (2016 update) [26]. Tumour size was assessed by a qualified pathologist. Moreover, we also evaluated the association between studied polymorphisms and the pathomorphological status of tumours. For this purpose, we divided BC patients into subgroups according to the diagnosis made by a specialist pathologist based on the 2004 World Health Organisation/International Society of Urological Pathology (WHO/ISUP) classification system for non-invasive urothelial neoplasia’s and infiltrating urothelial carcinomas [27]. Because the number of BC patients with urothelial and inverted papilloma and tumours infiltrating the muscle membrane was limited, these cases were omitted from the analysis. However, we did not detect any association between all studied SNPs of the *IL-6* and *IL-8* genes and TNM stage and histopathological status (Supplementary Table S4).

### 2.6. SNPs of IL-6 and IL-8 and BC Occurrence in the Male and Female Subpopulation

The epidemiological data indicate that men are much more likely to develop BC than women [1]. Therefore, we investigated an association between BC occurrence in male and female groups and all studied SNPs. However, we did not find any correlation between the genotypes/alleles of the studied SNPs and BC occurrence in the male and female subpopulations (Supplementary Table S5).

### 2.7. SNPs of Genes Encoding Interleukins and BC Occurrence in theNormal Body Weight/Overweight and Obesity Groups and in the Non-Smoker/Smoker Subpopulations

Cigarette smoking and being overweight or obese are recognised risk factors for BC development [8,28]. Therefore, we analysed frequency distributions of the genotypes of studied polymorphic variants between patients and controls in non-smoker/smoker subpopulations and the normal body weight/overweight and obesity subgroups. The distribution of such genotypes is shown in Supplementary Table S6 and S7. Unfortunately, we did not find any association between the genotypes/alleles of studied SNPs and BC occurrence in the analysed subgroups.

### 2.8. IL-6 and IL-8 mRNA Level Analysis

**Figure 2:**
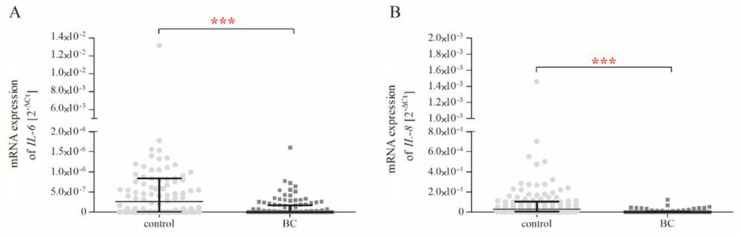
Basal mRNA expression of *IL-6* (**A**) and *IL-8* (**B**) genes in PBMCs of controls (n _control_ = 114) and patients with BC (n _patients with BC_ = 116). Basal gene expression levels were calculated using the 2^−ΔCt^ method (C_t gene_ − C_t 18S_). The data are plotted as individual values and the median with interquartile range is indicated by the horizontal bars; ***—*p* < 0.001.

The analysis of *IL-6* and *IL-8* expressions showed that patients with BC were characterised by lower (*p* < 0.001) mRNA levels of these genes compared to controls (Figure 2).

### 2.9. IL-6 and IL-8 Expression and Correlation with Size or the Direct Extent of the Primary Tumour, the Status of Lymph Node Metastasis, and Distant Metastasis according to the TNM Classification

**Figure 3:**
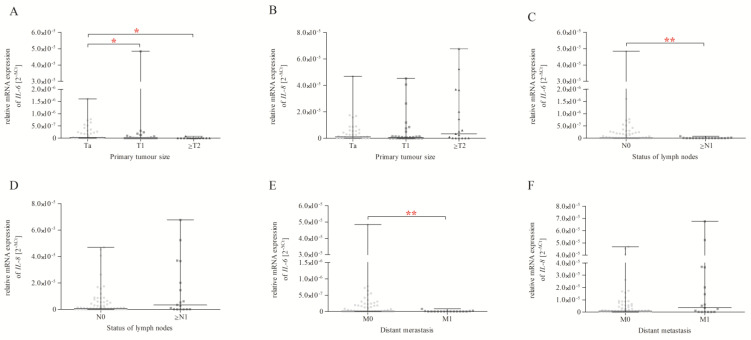
Basic *IL-6* and *IL-8* expression in primary tumour size groups (**A**,**B**), the status of lymph nodes (**C**,**D**), and distant metastasis (**E**,**F**), expressed using the 2^−ΔCt^ (C_t gene_ − C_t 18S_) method for each sample. The data are plotted as individual values and the median with interquartile range is indicated by the horizontal bars; *—*p* < 0.05; **—*p* < 0.01.

We assessed the impact of primary tumour size (Figure 3A,B), the status of lymph node metastasis (Figure 3C,D), and distant metastasis (Figure 3E,F) on the level of expression of the studied genes. For the purposes of analysis, we divided BC patients into subgroups according to the size of the primary tumour (Ta, T1, ≥T2), the status of lymph nodes (N0, ≥N1), and distant metastasis (M0, M1) according to the TNM classification [26]. We found that BC patients with Ta status primary tumour size were characterised by higher *IL-6* expression (Figure 3A) than BC patients with T1 and ≥T2 status (*p* < 0.05). In the case of lymph node metastasis and distant metastasis, our study confirmed that BC patients with N0 and M0 status showed higher *IL-6* expression (Figure 3C,E) than BC patients with ≥N1 and M1 status (*p* < 0.01). In the case of *IL-8* expression, we observed no statistical differences in the analysis of primary tumour size (Figure 3B), the status of lymph node metastasis (Figure 3D), and distant metastasis (Figure 3F).

### 2.10. IL-6 and IL-8 Expression and Correlation with the Grading of Histological Malignancy

We also evaluated the impact of the pathomorphological status of tumours on the level of *IL-6* and *IL-8* expression. For this purpose, as in the case of the SNP analysis, we divided BC patients into subgroups according to the diagnosis made by a specialist pathologist based on the 2004 WHO/ISUP classification system [27]. Because the number of BC patients with urothelial and inverted papilloma and tumours infiltrating the muscle membrane was limited, these cases were omitted from the analysis. Unfortunately, for both *IL-6* and *IL-8* expression levels, we detected no statistical differences between pathomorphological subgroups (Supplementary Figure S1).

### 2.11. IL-6 and IL-8 Expression in Genotype Subgroups

**Figure 4:**
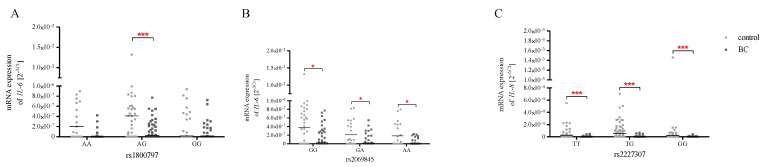
Relative *IL-6* (**A**,**B**) and *IL-8* (**C**) expression in PBMCs in the genotype groups of all studied SNPs, expressed using the 2^−ΔCt^ (C_t gene_ − C_t 18S_) method for each sample. The data are plotted as individual values and the median with interquartile range is indicated by the horizontal bars; *—*p* < 0.05; ***—*p* < 0.001.

Genetic variation in mRNA expression plays a crucial role in human phenotypic diversity. Phenotypic differences may result from genetic SNPs acting by altering the protein-coding sequence or at the RNA level, affecting transcription (activation or inhibition through regulatory sites or the structure of regulatory elements), mRNA processing, mRNA pre-splicing, exonic splicing enhancers (ESEs), exon skipping, and regulatory RNAs [29]. Therefore, we also investigated the association between mRNA expression and SNPs in the genotype groups. Unfortunately, this analysis showed no impact genotypes for each studied SNP in terms of mRNA expression of *IL-6* and *IL-8* (Supplementary Figure S2). On the other hand, additional analysis of *IL-6* and *IL-8* expression levels in the genotype groups confirmed the existence of significant differences between the control group and patients with BC (Figure 4). We found that heterozygotes of the -597 A>G (rs1800797) SNP were characterised by higher *IL-6* expression in controls than in BC patients (*p* < 0.001). Moreover, in the case of all genotypes of c.3331 G>A (rs2069845), polymorphism showed lower expression of *IL-6* in patients with BC than in the control group (*p* < 0.05). Similarly, in the case of all homozygotes and heterozygotes of c.+396 T>G (rs2227307) polymorphic variants, BC patients were characterised by decreased *IL-8* expression compared to healthy volunteers (*p* < 0.001).

### 2.12. Effect of Gender/BMI/Cigarette Smoking and BC on the mRNA Expression of IL-6 and IL-8

**Figure 5:**
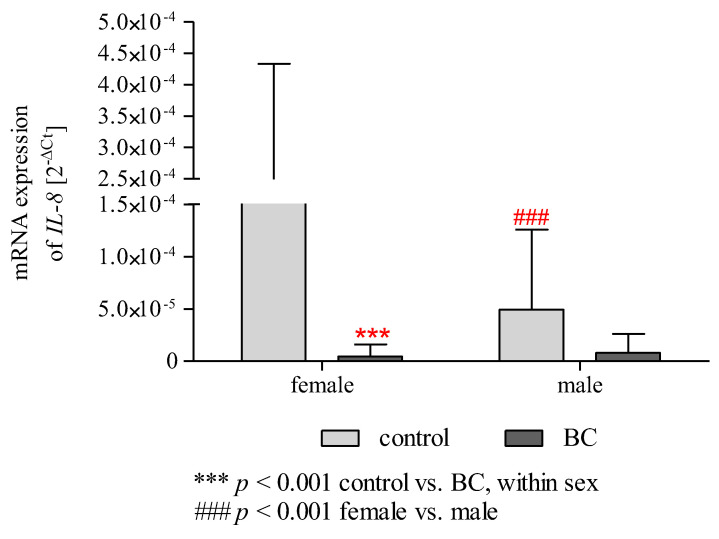
Two-way ANOVA with Bonferroni post hoc tests shows significant effects for gender and BC on the mRNA expression of *IL-8*. Gene expression in PBMCs is expressed using the 2^−ΔCt^ (C_t gene_ − C_t 18S_) method. The data are presented as mean ± SD.

Statistical analysis using two-way ANOVA (Table 3), which showed a difference between control and BC groups (*p* < 0.001), also indicates that gender has a significant effect in terms of *IL-8* expression level (*p* < 0.001). Additionally, two-way ANOVA analysis showed significant effects in terms of the interaction of gender × group for *IL-8* expression (*p* < 0.001). Two-way ANOVA with Bonferroni post hoc tests (Figure 5) showed that *IL-8* expression was significantly higher in a subgroup of women than in men among controls (*p* < 0.001). Moreover, patients with BC were characterised by significantly reduced expression of *IL-8* compared to the control group in a subgroup of women (*p* < 0.001). This relationship was not observed in men. In the case of BMI and cigarette smoking, we observed no impact on the mRNA expression of *IL-6* and *IL-8* (Table 3, Supplementary Figure S3).

### 2.13. The Methylation Status of the IL-6 Promoter Region

**Figure 6:**
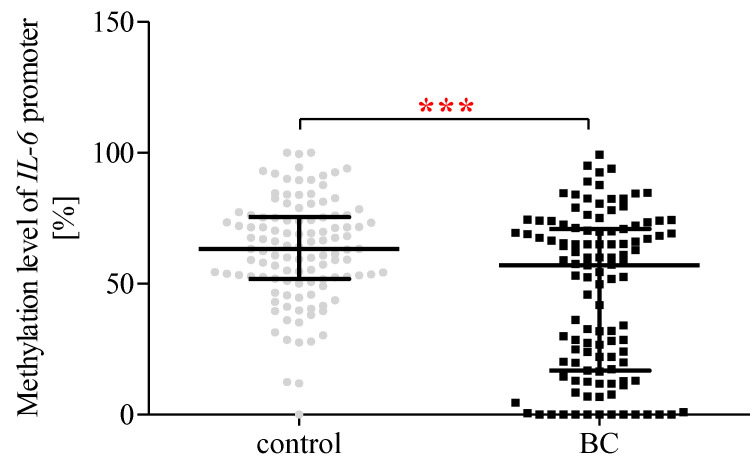
Methylation status of the *IL-6* promoter region in PBMCs. The data are plotted as individual values and the median with interquartile range is indicated by the horizontal bars; ***—*p* < 0.001.

Our analysis showed that patients with BC were characterised by a lower (*p* < 0.001) methylation level of the *IL-6* promoter region compared to the controls (Figure 6).

### 2.14. Methylation of the IL-6 Promoter Region and Correlation with Size or the Direct Extent of the Primary Tumour, the Status of Lymph Node Metastasis, Distant Metastasis According to the TNM Classification, and the Grading of Histological Malignancy

**Figure 7:**
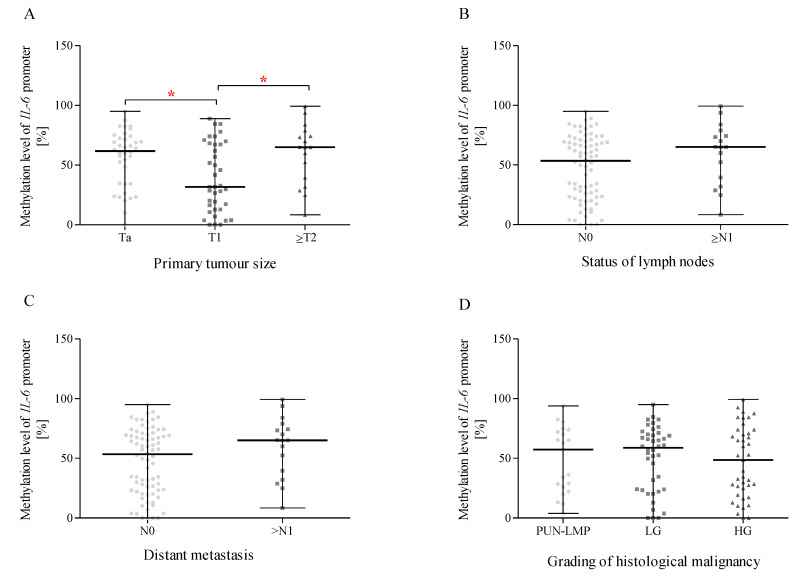
Methylation status of the *IL-6* promoter region in terms of primary tumour size groups (**A**), the status of lymph node metastasis (**B**), distant metastasis (**C**), and the grading of histological malignancy (**D**). The data are plotted as individual values and the median with interquartile range is indicated by the horizontal bars; *—*p* < 0.05.

We verified the impact of primary tumour size (Figure 7A), the status of lymph node metastasis (Figure 7B), distant metastasis (Figure 7C), and the grading of histological malignancy (Figure 7D) on the methylation level of the *IL-6* promoter region. For this purpose, similarly to the expression level analyses, we divided them into appropriate subgroups [26,27]. We found that BC patients with T1 status primary tumour size were characterised by lower methylation status of the *IL-6* promoter (Figure 7A) than BC patients with Ta and ≥T2 statuses (*p* < 0.05). In the case of lymph node metastasis (Figure 7B) and distant metastasis (Figure 7C), as well as the grading of histological malignancy (Figure 7D), our study showed no statistical differences in the methylation status of the *IL-6* promoter between analysed subgroups.

### 2.15. Effect of Gender/BMI/Cigarette Smoking and BC on the Methylation Level of the IL-6 Promoter Region

**Figure 8:**
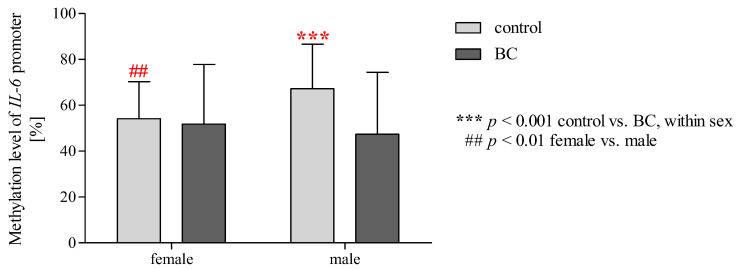
Two-way ANOVA with Bonferroni post hoc tests shows significant effects for gender and BC on the methylation level of the *IL-6* promoter. The data are presented as mean ± SD.

Statistical analysis using two-way ANOVA (Table 4) showed a difference between the control and BC groups (*p* < 0.01). Additionally, two-way ANOVA analysis showed significant effects for the interaction of gender × group in terms of the methylation status of the *IL-6* promoter region (*p* < 0.05). Two-way ANOVA with Bonferroni post hoc tests (Figure 8) showed that the methylation level of the *IL-6* promoter was significantly higher in a subgroup of men than in women among controls (*p* < 0.05). Moreover, patients with BC were characterised by the significantly reduced methylation status of the *IL-6* promoter compared to the control group in a subgroup of men (*p* < 0.001). This relationship was not observed in women. In the case of BMI and cigarette smoking, we observed no impact on the methylation status of the *IL-6* promoter region (Table 4, Supplementary Figure S4).

## 3. Discussion

Chronic persistent inflammation can contribute to cell damage, promoting the development of many diseases, including cancer. Virchow initially put forward a hypothesis regarding the location of inflammation in the region of cancer development in 1863, emphasising the role of prolonged irritation, tissue damage, and the activated local reaction of the host in cell proliferation [30]. Although it is now known that the essential proliferation of cells does not cause cancer, it is also clear that cell proliferation in an environment rich in inflammatory cells, growth factors, increased angiogenesis, and DNA damage factors can ensure the conditions needed for the formation and progression of cancer. Previous studies confirm that cancer development is preceded by chronic inflammation in up to a third of all cases [31]. However, thorough molecular disorders leading to malignant transformation in the chronic inflammation environment have not been fully explained. Nevertheless, among factors attributed to cancer transformation, pro-inflammatory cytokines are seen, including IL-6 and IL-8.

IL-6 is a glycoprotein consisting of 184 amino acids with a molecular weight of 26 kDa, while IL-8 is a non-glycosylated polypeptide composed of 72 amino acids with a total mass of 8452 Da [32]. IL-6 is a pleiotropic cytokine involved in a wide range of physiological processes, such as organ development, acute phase response, inflammation, immune responses, and metabolic regulation, among others [33]. The cytokine mainly activates the JAK1/STAT3 (Janus Kinase 1/signal transducer and transcription activator 3) signal trail and acts as a pro-inflammatory factor. Moreover, a high level of IL-6 can favour humoral immune response T-Helper-2 (Th2), which does not contribute to combating cancer [34]. In turn, IL-8 is not only responsible for attracting neutrophils to sites of inflammation, but also for promoting the growth and differentiation of monocytes and macrophages [35], endothelial cell survival, proliferation, and angiogenesis [36]. In addition, IL-8 also increases oxidative metabolism and the production of reactive oxygen species, which probably leads to oxidative stress [37]. Notably, IL-6 and IL-8 are crucial for epithelial–mesenchymal transition (EMT) via activation of the protein kinase activated by the mitogen (MAPK) pathway, with these key processes promoting cell motility, wound healing, tissue regeneration, fibrogenesis, and tumour metastasis [38,39].

The modification of the pathways involving IL-6 and IL-8 observed during carcinogenesis may be the result of polymorphisms of genes encoding these proteins [29]. Nevertheless, previous studies have focused primarily on the analysis of environmental risk factors for the development of bladder cancer, including obesity, smoking, and exposure to aromatic amines [1,2,3,4,5,6,7,8,9,10]. Consequently, knowledge about the potential role of *IL-6* and *IL-8* gene polymorphisms and other changes at the molecular level and their relationship to BC development is incomplete. Therefore, our research aimed to assess the impact of -597 A>G (rs1800797), c.3331 G>A (rs2069845), and c.+396 T>G (rs2227307) SNP occurrence on the frequency of BC occurrence and the impact of BC on the level of expression and the degree of methylation of the promoter regions of the studied genes. Previous studies have focused on determining the relationship between the *IL-6*-174G/C polymorphism (rs1800795) and bladder cancer. Obtained results confirmed that the C/C genotype of the -174G/C SNP was associated with an increased risk of BC in ever-smokers but not never-smokers. In addition, heavy smokers with the C/C *IL-6* genotype were more likely to develop BC than light smokers. Interestingly, in patients with nonmuscle-invasive bladder cancer, the C/C genotype of the -174G/C SNP was associated with an increased risk of BC. In turn, in patients with invasive BC, the same genotype was correlated with the improvement of a 5-year total and specific experience for the disease [29,40,41]. In the case of *IL-8*, the specific analysed polymorphism is −251 T/A (rs4073). Wu et al. (2013) found that patients with the T/T genotype were characterised by an increased risk of BC development [42]. On the other hand, Leibovici’s team did not find significant associations between the −251 T/A (rs4073) SNP of *IL-8* and BC risk [38]. However, another study showed that people who had smoked before and who were carriers of the T/T genotype were characterised by a significantly increased BC risk compared to people who had never smoked and who were carriers of the T/A or A/A genotype [43]. In our study, we assessed the effect of two polymorphisms of the *IL-6* gene (-597 A>G (rs1800797) and c.3331 G>A (rs2069845)) and one of the *IL-8* gene (c.+396 T>G (rs2227307)) on the incidence of BC. Although all tested SNPs are located within introns, each was associated with the severity of inflammation [22,44,45]. Unfortunately, despite previous studies indicating the role of increased inflammatory processes in the process of neoplastic transformation, we did not find a correlation between the genotypes/alleles of all polymorphisms and the occurrence of BC in this study, even in the subgroup of smokers.

Additionally, in our study, we also analysed mRNA expression of *IL-6* and *IL-8* and the methylation status of the *IL-6* promoter in PBMCs of patients with BC and controls. We found a decreased level of *IL-6* expression in patients with BC compared to controls. On the other hand, in vitro study showed that healthy primary bladder fibroblasts induced by bladder cancer-derived exosomes showed higher *IL-6* expression than healthy primary bladder fibroblasts [23]. Similarly, Chen’s team detected that the BC tissue samples were characterised by higher *IL-6* expression than normal tissue samples taken from the same six patients [46]. These discrepancies between the results of our and previous studies may be the result of the diversity of the material that was analysed. Our research focuses on the search for potential molecular biomarkers enabling early diagnosis of BC. Therefore, the starting material for our experiments was blood, which is a relatively readily available material. Other cited studies were based on genetic material obtained from cell cultures or directly from the tissue of bladders changed by cancer. Thus, it is possible that the changes occurring centrally (directly in the transformed tissue) do not correspond to the changes observed peripherally in PBMCs. Therefore, further research is needed to compare the molecular changes occurring within the tumour and PBMCs. Interestingly, our analysis also showed the effect of gender on the level of *IL-6* and *IL-8* expression. Both in the control group and BC patients, a higher level of expression was observed in the female population than in the male population; however, this difference was significant only for the control group in the case of *IL-8* expression. Previous studies have confirmed that the lower expression of *IL-6* and *IL-8* in women than in men is the result of the effect of estradiol-17β on macrophages, which inhibits the NF-κB pathway. NF-κB induces the expression of proteins that trigger the inflammatory response, such as cytokines, chemokines, and cell adhesion molecules. The 7β-estradiol-bound estrogen receptor blocks DNA binding and the transcriptional activity of p65 (one of the five components that form NF-κB), preventing its nuclear translocation. The lack of p65 translocation to the nucleus prevents activation of the transcription of pro-inflammatory factors such as IL-6. Similarly, estradiol-17β may contribute to *IL-8* down-expression. In this case, the 7β-estradiol-bound estrogen receptor inhibits IKK activity and causes IκB protein degradation, blocking DNA binding by NF-κB. Consequently, this leads to a reduction in the intensity of the inflammatory response during the course of various diseases, including cancer [47,48,49]. Moreover, we also observed a significant increase in the expression of *IL-6* in the case of the T1 stage compared to the Ta stage. The relation between higher *IL-6* expression and unfavourable prognostic characteristics could be related to the fact that *IL-6* is crucial for tumour progression from the Ta stage into the T1 stage. Previous studies on the role of cytokines in BC progression have shown that *IL-6* binding to its receptor (IL-6R) induces the phosphorylation of signal transducer and transcription activator 3 (STAT3), which undergoes dimerisation and translocation to the nucleus to regulate transcription EMT genes, including E-cadherin, phospho-ß-catenin and phospho-GSK3ß, N-cadherin, and vimentin. Thus, the upregulation of IL-6 may be a crucial step in BC progression and metastasis [50]. On the other hand, in the case of patients with a stage ≥T2, downregulation of *IL-6* expression levels was observed in our analysis, with levels below even those of Ta patients. Due to the differentiation of results for the analysed subgroups, it is necessary to extend the studies to assess expression in each possible tumour stage subgroup (from Ta to T3).

In the case of *IL-8*, underexpression was observed in cancerous bladder tissue. On the other hand, the increased expression of *IL-8* was correlated with a poor prognosis of BC [51]. Moreover, high levels of IL-8 secretion in peripheral blood leukocytes was significantly associated with shorter recurrence-free survival in patients with bladder cancer receiving Bacillus Calmette–Guerin (BCG) therapy. Similarly, high baseline urinary IL-8 levels were also predictive of shorter time to tumour recurrence in NMIBC patients [47]. However, in the presented study, we found significantly decreased *IL-8* expression in BC patients compared to controls. Moreover, we confirmed that the mRNA level of *IL-8* was higher in patients with high-grade and infiltrative tumours, but this association turned out to be statistically insignificant. The association between higher *IL-8* expression and unfavourable prognosis may be due to the significant role of *IL-8* in the progression, not induction, of tumour development. The increased level of *IL-8* expression observed in tumour tissue at the mRNA and protein level promotes the recruitment of B lymphocytes to the location of BC cells. In turn, in the vicious circle mechanism, infiltrating cells may further increase the level of IL-8 in the tumour microenvironment, which may play a key role in tumour progression due to their ability to enhance tumour proliferation, invasion, and angiogenesis by inducing the expression of metalloproteinases [52].

What is more, we were the first to assess the impact of BC development on the degree of methylation of the *IL-6* promoter region. Interestingly, the methylation level of this region is lower in the general patient population compared to controls, suggesting increased expression in BC patients. This was not confirmed by our obtained results. These discrepancies between the expected and obtained directions of expression based on methylation status (the presence of a high level of *IL-6* mRNA expression and a high degree of methylation of the *IL-6* promoter) could suggest that gene expression is subject to other forms of regulation than the methylation of promoter regions. Other known epigenetic modifications that alter DNA accessibility and chromatin structure, thereby regulating patterns of gene expression, could also be considered, such as histone modification (methylation and acetylation) and nucleosome positioning [53]. However, among BC patients, taking into account the stage of cancer, we confirmed that the lowest degree of methylation was found in patients from the T1 subgroup, which corresponded to the highest level of *IL-6* expression.

In conclusion, we did not confirm the hypothesis that chronic inflammation plays a crucial role in the pathogenesis of BC. The polymorphisms located in the *IL-6* and *IL-8* genes that were selected for our analysis did not significantly affect the frequency of BC in the Polish population, and we additionally noted that patients with BC were characterised by decreased levels of expression and methylation of the studied genes. Although obtained results are inconsistent with previous data, this may suggest high BC heterogeneity. Moreover, it should be underlined that our study had several limitations. First, we examined only a few selected candidate polymorphisms that had not yet been analysed, so it seems necessary to genotype more SNPs in inflammatory genes and analyse haplotypes to confirm and extend our findings. Second, our research is limited to one ethnic population and includes relatively small sample sizes, which, in turn, leads to the possibility of not repeating the results in other populations. In addition, there is a justified need for further research to more accurately determine the risk associated with the level of *IL-6* and *IL-8* expression and methylation for each stage of the disease, which will consequently make it possible to determine whether a pharmacological intervention aimed at modulating the level of *IL-6* and *IL-8* will have a therapeutic role in the treatment of BC. The presented limitations result from the specificity of the analysed material and its limited availability. Therefore, further replication studies performed on other ethnic populations are needed to verify the conclusion that chronic inflammation is the cause of BC.

## 4. Materials and Method

### 4.1. Participants

The case–control study consisted of 230 participants from Poland, including 116 patients with BC (32 women and 84 men; mean age 69.67 ± 11.26) confirmed by histopathological examination and 114 age- and sex-matched healthy volunteers without BC who had no family history of BC (control group: 39 women and 75 men; mean age 66.71 ± 11.76). Patients were recruited from the Department of Urology at the Provincial Integrated Hospital in Plock, Poland, from 30 March 2021 to 31 December 2022 and selected randomly without replacement sampling. Moreover, all of the participants were genetically unrelated individuals. Recruitment of patients consisted of conducting a short interview with the medical coordinator of the study, a urologist. During the interview, the medical coordinator briefly explained the study’s aims and that participation would require a collection of epidemiological, clinical, and anatomopathological data and future whole blood collection by specialised hospital staff. In the selection process of BC patients, exclusion criteria included age below 18, previous or current neoplastic diseases other than BC, autoimmune disorders, or the refusal to consent to participate in the study. Patients and controls who agreed to participate in the study were asked for signed informed consent. All study participants then completed a structured questionnaire to identify demographic and potential BC risk factors. Patients and healthy volunteers provided information on their age, lifestyle habits (e.g., daily fluid intake including coffee, type of diet), smoking, body mass index (BMI), and comorbidities (e.g., hypertension, blood pressure, diabetes, hypercholesterolaemia). Where possible, the validity and reliability of the questionnaires was checked. Detailed characteristics of BC patients and the control group are presented in Table 1. In addition, patients with suspected BC underwent histopathological examination. Histological reviews of tumours were performed and confirmed by a trained pathologist in urological oncology based on the 2004 World Health Organisation/International Society of Urological Pathology (WHO/ISUP) classification system of non-invasive urothelial neoplasia and infiltrating urothelial carcinomas [27]. The clinical tumour stage was determined according to the 8th edition of the TNM Classification of Malignant Tumours developed by the Union for International Cancer Control (UICC) and used by the American Joint Committee on Cancer (AJCC) and the International Federation of Gynaecology and Obstetrics (FIGO) (2016 update) [26]. All studied methods were carried out according to the declaration of Helsinki and performed in line with relevant guidelines and regulations. The project was approved by the Bioethics Committee of the Faculty of Biology and Environmental Protection at the University of Lodz, Poland (approval no. 12/KBBN-UŁ/II/2020–21), and the Bioethics Committee of the Medical University of Lodz (no. RNN/141/21/KE).

### 4.2. Blood Sample Collection and DNA and RNA Isolation

**Figure 9:**
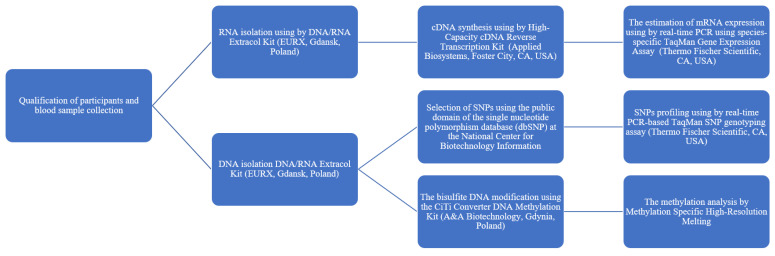
The course of planned experiments.

About 4 mL of venous blood from BC patients and controls (after qualifying for participation in the study) was collected into BD Vacutainer^®^ EDTA tubes (Becton, Dickinson and Company Sparks, Sparks, MD, USA), coded, and stored at −20 °C until further use (Figure 9). Genomic DNA and total RNA samples were isolated from frozen peripheral blood using a DNA/RNA Extracol Kit (EURX, Gdansk, Poland) according to the manufacturer’s instructions. In the next step, concentrations of the isolated DNA and RNA samples were determined by spectrophotometric measurement of absorbance at 260 nm, and purities were calculated by A260/A280 ratio using a Bio-Tek Synergy HT Microplate Reader (Bio-Tek Instruments, Winooski, VT, USA).

### 4.3. Selection of SNPs and Genotyping

We selected three polymorphisms (Table 5), namely -597 A>G (rs1800797) and c.3331 G>A (rs2069845) in *IL-6* and c.+396 T>G (rs2227307) in *IL-8*, using the public domain of the single nucleotide polymorphism database (dbSNP) at the National Centre for Biotechnology Information (NCBI, http://www.ncbi.nlm.nih.gov/snp; accessed on 1 January 2022) and the available literature. The minor allele frequency (MAF) of all chosen SNPs was higher than 0.05 in the European population (submitter population ID: HapMap-CEU). Moreover, the selected polymorphisms have a confirmed impact on the course of immune response, the disturbances of which also are observed in the development of BC [20,21,22]. Analysis of the available literature also confirmed the influence of the selected polymorphisms on the incidence of numerous cancers, including prostate cancer, breast cancer, cervical cancer, rectal cancer, colon cancer, and gallbladder cancer [54,55,56,57,58,59].

**Figure 10:**
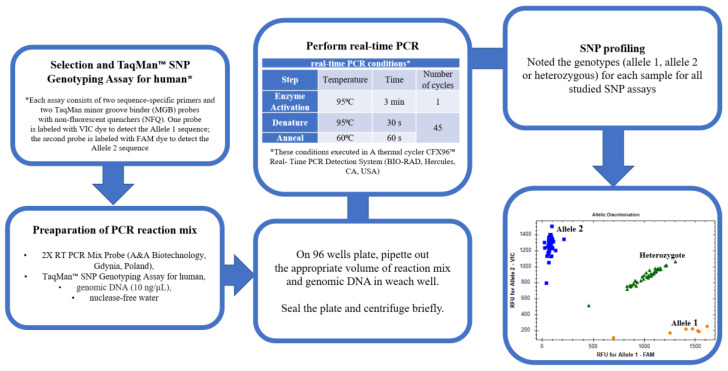
Detailed steps for real-time PCR of SNP profiling.

SNP profiling was performed using a real-time PCR-based TaqMan SNP genotyping assay (Thermo Fischer Scientific, Carlsbad, CA, USA) and RT PCR Mix Probe (A&A Biotechnology, Gdynia, Poland) according to the manufacturer’s recommendations on a CFX96™ Real-Time PCR Detection System Thermal Cycler (Bio-Rad Laboratories, Inc., Hercules, CA, USA). Detailed steps of 6-well reaction plate preparation and the conditions of real-time PCR are elucidated in Figure 10.

### 4.4. cDNA Synthesis and mRNA Expression Levels

*IL-6* and *IL-8* gene expression was determined by real-time PCR using a species-specific TaqMan gene expression assay (*IL-6*—assay ID Hs00174131_m1; *IL-8*—assay ID Hs00174103_m1; *18S* as reference gene—assay ID Hs99999901_s1; Thermo Fisher Scientific, Waltham, MA, USA) and RT PCR Mix Probe (A&A Biotechnology, Gdynia, Poland) according to the manufacturer’s instructions on a CFX96™ Real-Time PCR Detection System Thermal Cycler (Bio-Rad Laboratories, Inc., Hercules, CA, USA). Before real-time PCR, cDNA was synthesised using total RNA and an Applied Biosystems High-Capacity cDNA Reverse Transcription Kit (Foster City, CA, USA) according to the manufacturer’s protocols. Finally, the level of mRNA expression was measured in relation to that of the reference gene (18S ribosomal RNA as an internal mRNA control) and relative mRNA expression levels were calculated using the 2^−ΔCt^ method [60].

### 4.5. Bisulphite Treatment and Methylation Analysis by MS-HRM

Genomic DNA was processed for bisulphite DNA modification using the CiTi Converter DNA Methylation Kit (A&A Biotechnology, Gdynia, Poland) according to the manufacturer’s protocol. This step makes it possible to distinguish between a methylated CpG island and an unmethylated one because the bisulphite conversion is a procedure where the DNA is denatured and delamination of unmethylated cytosines into uracils occurs after sodium bisulphite treatment, whereas methylated cytosines stay unaffected. Quantitative detection of CpG methylated islets was performed by methylation-specific high-resolution melting (MS-HRM) using the Bio-Rad CFX96 Real-Time PCR Detection System (BioRad Laboratories Inc., Hercules, CA, USA) equipped with Bio-Rad Precision Melt Analysis Software (BioRad Laboratories Inc., Hercules, CA, USA) to analyse methylation status. For this purpose, specific primers were designed to assess *IL-6* promoter methylation using MethPrimer (https://www.urogene.org/cgi-bin/methprimer/methprimer.cgi; accessed on 1 December 2022) according to the recommendations of Wojdacz et al. (2009) [61]. In the case of *IL-8*, the status of methylation cannot be assessed due to the absence of CpG islands within the promoter region. The primers for analysing *IL-6* (RefSeq: NM_001318095) methylation were the following: forward, 5′-TTATGTAGGAAAGAGAATTTGGTTTAG-3′; reverse, 5′-AAAAAATAAAATCATCCATTCTTCAC-3′. MS-HRM analyses consisted of the following three main steps: initial activation (3 min at 95 °C) proceeded by 45 cycles of 95 °C for 30 s; annealing at optimal primer temperatures (tested experimentally—61.4 °C) for 60 s; and elongation at 72 °C for 60 s leading to the last step of the melting curve. HRM analysis consisted of denaturation at 95 °C for 15 s, reannealing at 60 °C for 1 min, and melting from 60 to 95 °C at a ramp rate of 0.2 °C. All reaction samples contained RT PCR Mix EvaGreen^®^ (A&A Biotechnology, Gdynia, Poland), 500 NM of forward and reverse primers, bisulphite-modified DNA template (10 ng/µL), and PCR-grade water. Finally, methylation status was evaluated based on HRM profiles obtained from the amplification of methylated template DNA (CpGenome Human Methylated DNA Standard Set, Merck Millipore Burlington, MA, USA) and unmethylated DNA (CpGenome Human Non-Methylated DNA Standard Set, Merck Millipore Burlington, MA, USA). Therefore, serial dilutions of template DNA were prepared: 0%, 10%, 25%, 50%, 75%, and 100% methylated DNA.

### 4.6. Statistical Analysis

All statistical analyses were performed using Statistica 12 (Statsoft, Tulsa, OK, USA) and SigmaPlot 11.0 (Systat Software Inc., San Jose, CA, USA). The chi-square test was used to assess Hardy–Weinberg equilibrium of the observed genotype frequencies with the expected frequencies among the case and control subjects. The association between genotypes and BC was evaluated by computing the odds ratios (ORs) and 95% confidence intervals (95% CI) from unconditional multiple logistic regression analyses (codominant, dominant, and recessive models). In addition, because men suffer from BC much more often than women, the ORs were adjusted for gender [1]. Moreover, we also used an unconditional logistic regression model to evaluate the correlation between cases and controls for each studied polymorphism in the male/female population, non-smoker/smoker groups, and subpopulations in the normal body weight/overweight/obesity groups. We then also performed the synergy factor (SF) analysis proposed by Mario Cortina-Borja et al. (2009), which was used to confirm potential SNP–SNP interactions and associations with BC [25]. Linkage disequilibrium (LD) and haplotype distribution were evaluated based on the known genotypes of two polymorphisms (rs1800797, rs2069845) using SHEsisPlus software (http://shesisplus.bio-x.cn/SHEsis.html, accessed on 23 December 2022) [62]. In addition, the Pearson’s χ2 test was used to compare the frequency distributions of various clinical characteristics of the different genotypes of all studied polymorphisms. For analysis of mRNA expression and the demographics and baseline characteristics of patients, the Shapiro–Wilk test was used for the examination of distribution normality. The significance of the difference between studied values was then determined based on the Mann–Whitney test or Student’s *t*-test. Moreover, the Kruskal–Wallis One Way Analysis of Variance on Ranks test was used to compare relative mRNA expression between groups of genotypes. Evaluation of the effects of gender/BMI/cigarette smoking and BC on mRNA expression were analysed using two-way ANOVA analyses. Finally, the Bonferroni test was used as a post hoc test. Values of *p* < 0.05 were considered statistically significant.

## Figures and Tables

**Table 1:** Clinical demographic characteristics of study participants.

Demographic Characteristics of the Study Participants					
Feature			Controls (*n* = 114) *Frequency*	Patients with BC (*n* = 116) *Frequency*	*p* *
Gender	females		0.34	0.28	0.278
	males		0.66	0.72	
**Age**	**mean ± SD**		**66.71 ± 11.76**	**69.67 ± 11.26**	**0.031**
	**range**		**28–91**	**20–92**	
Education	primary (basic) education		0.16	0.30	0.087
	vocational education		0.39	0.25	
	high school education		0.33	0.42	
	university degree		0.12	0.03	
Residency	village		0.36	0.46	0.805
	a city with a population under 50 thou. residents		0.39	0.16	
	a city with a population over 50 thou. residents		0.25	0.39	
**Marital status**	**single**		**0.25**	**0.13**	**0.003**
	**married**		**0.67**	**0.70**	
	**widow/widower**		**0.08**	**0.17**	
**Professional activity**	**physical work**		**0.28**	**0.20**	**0.031**
	**mental work**		**0.17**	**0.05**	
	**unemployment**		**0.01**	**0.04**	
	**pension**		**0.64**	**0.71**	
Smoking	never		0.56	0.31	0.367
	former		0.24	0.38	
	current		0.20	0.31	
BMI [kg/m^2]^	mean ± SD		27.69 ± 3.51	27.12 ± 4.83	0.305
	<25		0.31	0.36	
	25–30		0.42	0.37	
	>30		0.27	0.27	
Daily fluid intake	<2 L/day		0.52	0.50	0.791
	>2 L/day		0.48	0.50	
Daily coffee consumption (number of cups, one cup has a capacity of 200 mL)	0		0.21	0.27	0.369
	1		0.44	0.41	
	2–3		0.32	0.30	
	>4		0.03	0.02	
**Total blood count of the study participants**					
Feature/Parameters			Controls (*n* = 114) *mean* ± *SD*	Patients with BC (*n* = 116) *mean* ± *SD*	*p*
**Red blood cells—RBC (×10^12^/L)**			**4.49 ± 0.61**	**4.29 ± 0.71**	**0.039**
**Haematocrit—HCT (%)**			**40.91 ± 5.26**	**38.80 ± 6.58**	**0.014**
**Haemoglobin—HGB (g/L)**			**13.56 ± 2.44**	**12.91 ± 2.33**	**0.030**
Mean corpuscular volume—MCV (fL)			91.25 ± 5.34	90.49 ±5.97	0.135
Mean cell haemoglobin—MCH (pg/cell)			30.20 ± 3.20	30.24 ± 2.74	0.290
Mean corpuscular haemoglobin concentration—MCHC (g/L)			33.27 ± 1.22	33.11 ±1.52	0.207
**Red cell distribution width—RDW (%)**			**13.50 ± 1.49**	**13.90 ± 1.32**	**0.010**
Haemoglobin distribution width—HDW (g/L)			2.53 ± 0.25	2.56 ± 0.40	0.467
**White blood cells—WBC (×10^9^/L)**			**9.23 ± 18.67**	**8.88 ± 5.07**	**0.032**
%HYPO			2.34 ± 4.23	3.63 ± 7.10	0.157
%MIKRO			1.35 ± 2.76	1.32 ± 2.56	0.903
%MAKRO			1.44 ± 1.85	1.77 ± 4.05	0.978
%HYPER			0.73 ± 0.54	0.72 ± 0.85	0.091
Blood platelets—PLT (×10^9^/L)			234.95 ± 70.80	259.25 ± 106.83	0.127
Mean platelet volume—MPV (fL)			8.68 ± 1.53	8.98 ±1.04	0.081
**Blood biochemical parameters**					
Feature/Parameters			Controls (*n* = 114) *mean* ± *SD*	Patients with BC (*n* = 116) *mean* ± *SD*	*p*
**Glucose (mmol/L)**			**5.92 ± 2.16**	**6.64 ± 2.29**	**<0.001**
**Creatinine (µmol/L)**			**106.94 ± 12.14**	**127.02 ± 156.58**	**0.005**
Sodium (mmol/L)			139.93 ± 2.75	139.48 ± 4.25	0.391
**Potassium (mmol/L)**			**5.57 ± 9.42**	**4.60 ± 0.56**	**0.043**
**Coagulation panel**					
Prothrombin time (s)			12.68 ± 2.48	12.19 ± 1.20	0.285
Prothrombin index (%)			92.90 ± 11.89	95.30 ± 8.59	0.237
International normalised ratio (INR)			1.13 ± 0.23	1.09 ± 0.11	0.883
Activated partial thromboplastin time (APTT, s)			30.67 ± 3.91	30.00 ± 3.11	0.609
Fibrinogen (mg/L)			400.24 ± 145.26	426.59 ±178.41	0.362
**Dipstick urinalysis**					
Feature/Parameters			Controls (*n* = 114) *mean* ± *SD*	Patients with BC (*n* = 116) *mean* ± *SD*	*p*
pH			5.84 ± 0.88	5.89 ± 1.01	0.911
Specific gravity			1.02 ± 0.01	1.02 ± 0.01	0.842
Feature/Parameters			Controls (*n* = 114) *Frequency*	Patients with BC (*n* = 116) *Frequency*	*p*
WBC	absence per high-power field		0.68	0.45	0.077
	single per high-power field		0.29	0.25	
	numerous per high-power field		0.04	0.30	
Nitrite	negative		0.88	0.88	0.658
	positive		0.12	0.12	
Glucose	negative		0.98	0.95	0.262
	positive		0.02	0.05	
**Protein**	**negative**		**0.76**	**0.44**	**<0.001**
	**positive**		**0.24**	**0.56**	
Ketones	negative		0.90	0.90	0.964
	positive		0.10	0.10	
**Bilirubin**	**negative**		**0.98**	**0.89**	**0.006**
	**positive**		**0.02**	**0.11**	
Urobilinogen	normal level		1.00	0.94	0.711
	above normal		0.00	0.06	
Colour	pale yellow		0.05	0.11	0.218
	straw/yellow		0.82	0.63	
	dark yellow		0.02	0.02	
	amber		0.05	0.10	
	brown		0.01	0.06	
	red		0.04	0.08	
**Clarity**	**clear**		**0.74**	**0.48**	**<0.001**
	**slightly cloudy**		**0.12**	**0.25**	
	**cloudy**		**0.06**	**0.07**	
	**very cloudy**		**0.08**	**0.20**	
**Urine microscopy**					
Feature/Parameters			Controls (*n* = 114) *Frequency*	Patients with BC (*n* = 116) *Frequency*	*p*
**RBC**	**0–3/high power field**		**0.78**	**0.33**	**<0.001**
	**3–5/high power field**		**0.07**	**0.03**	
	**5–10//high power field**		**0.03**	**0.12**	
	**10–15/high power field**		**0.03**	**0.22**	
	**15–20/high power field**		**0.03**	**0.04**	
	**20–25/high power field**		**0.06**	**0.26**	
**WBC**	**1–3/high power field**		**0.66**	**0.45**	**0.006**
	**3–5/high power field**		**0.05**	**0.14**	
	**5–10/high power field**		**0.19**	**0.20**	
	**10–15/high power field**		**0.01**	**0.05**	
	**20–25/high power field**		**0.08**	**0.15**	
Squamous epithelial cells	single per high power field		0.82	0.69	0.177
	sparse per high power field		0.10	0.23	
	many per high power field		0.08	0.08	
Mucus thread	single per high power field		0.00	0.38	0.851
	sparse per high power field		0.80	0.24	
	many per high power field		0.20	0.38	
**Bacteria**	**lack of/single per high power field**		**0.65**	**0.01**	**<0.001**
	**sparse per high power field**		**0.27**	**0.39**	
	**many per high power field**		**0.08**	**0.60**	
**Additional information**					
Feature/Parameters			Controls (*n* = 114) *Frequency*	Patients with BC (*n* = 116) *Frequency*	*p*
Comorbidities	**hypertension**	**yes**	**0.53**	**0.31**	**<0.001**
		**no**	**0.47**	**0.69**	
	diabetes	yes	0.17	0.16	0.955
		no	0.83	0.84	
	**hypercholesterolaemia**	**yes**	**0.22**	**0.10**	**0.017**
		**no**	**0.78**	**0.90**	
Family history of bladder cancer	yes		n/d	0.10	n/d
	no		n/d	0.90	
Actual therapy for bladder cancer	surgical treatment—TURBT (transurethral resection of bladder tumour)		n/d	0.91	n/d
	chemotherapy		n/d	0.03	
	cystectomy		n/d	0.06	
**Symptoms accompanying BC**					
Haematuria with clots	yes		n/d	0.68	n/d
	no		n/d	0.32	
Dysuria	yes		n/d	0.65	n/d
	no		n/d	0.35	
Recurrent urinary tract infections	yes		n/d	0.63	n/d
	no		n/d	0.37	
Pollakiuria	yes		n/d	0.61	n/d
	no		n/d	0.39	
Urgent pressures	yes		n/d	0.68	n/d
	no		n/d	0.32	
The feeling of something left behind after voiding	yes		n/d	0.08	n/d
	no		n/d	0.92	
Waiting for micturition	yes		n/d	0.05	n/d
	no		n/d	0.95	
Urinary incontinence problem	yes		n/d	0.54	n/d
	no		n/d	0.46	
Lower abdominal pain	yes		n/d	0.51	n/d
	no		n/d	0.49	
Weight loss	yes		n/d	0.41	n/d
	no		n/d	0.59	
Zubrod’s fitness level (ECOG scale)	0		n/d	0.84	n/d
	1		n/d	0.09	
	2		n/d	0.02	
	3		n/d	0.04	
	4		n/d	0.01	
	5		n/d	0	
TNM Classification of Malignant Tumours (TNM)	Tx		n/d	0.01	n/d
	T0		n/d	0.01	
	Ta		n/d	0.38	
	Tis		n/d	0.01	
	T1		n/d	0.41	
	T2		n/d	0.17	
	T3		n/d	0.01	
	T4		n/d	0.00	
Status of regional lymph nodes	N0–N1		n/d	0.82	n/d
	≥N2		n/d	0.18	
The presence of distant metastases	M0		n/d	0.82	n/d
	M1		n/d	0.18	
Pathomorphology of nonmuscle-invasive tumours	urothelial papilloma		n/d	0.02	n/d
	inverted papilloma		n/d	0.00	
	papillary urothelial neoplasm of low malignant potential (PUN-LMP)		n/d	0.16	
	low-grade papillary urothelial carcinoma (LG)		n/d	0.42	
	high-grade papillary urothelial carcinoma (HG)		n/d	0.36	
Pathomorphology of tumours infiltrating the muscle membrane	invasive urothelial carcinoma		n/d	0.00	n/d
	squamous cell carcinoma		n/d	0.04	
	glandular carcinoma		n/d	0.00	
	small cell carcinoma		n/d	0.00	
	undifferentiated carcinoma		n/d	0.00	
	other		n/d	0.02	

* *p* < 0.05 are in bold.

**Table 2:** Distribution of genotypes and alleles of the 597 A>G—*IL-6* (rs1800797), c.3331 G>A—*IL-6* (rs2069845), and c.+396 T>G—*IL-8* (rs2227307) polymorphisms and odds ratios (ORs) with confidence intervals (95% CIs) in patients with BC and controls.

Genotype/Allele	Control (*n* = 114)		BC (*n* = 116)		Crude OR (95% CI) *	*p*	Adjusted OR (95% CI) *	*p*
	Number	Frequency	Number	Frequency				
**-597 A>G—*IL-6* (rs1800797)**								
*Frequencies*								
A/A	25	0.219	24	0.207	0.929 (0.494–1.746)	0.818	0.905 (0.480–1.707)	0.757
A/G	57	0.500	61	0.526	1.109 (0.661–1.861)	0.695	1.113 (0.663–1.870)	0.685
G/G	32	0.281	31	0.267	0.935 (0.523–1.669)	0.819	0.950 (0.531–1.701)	0.864
χ^2^ = 230.000; *p* = 0.432								
A	107	0.469	109	0.470	1.002 (0.691–1.454)	0.991	0.986 (0.679–1.433)	0.942
G	121	0.531	123	0.530	0.998 (0.688–1.447)	0.991	1.014 (0.698–1.474)	0.942
*Carriage rates*								
A (+)	81	0.355	85	0.366	1.117 (0.627–1.989)	0.707	1.101 (0.617–1.965)	0.744
A (−)	33	0.145	31	0.134	0.895 (0.503–1.594)	0.707	0.908 (0.509–1.610)	0.744
G (+)	91	0.399	92	0.397	0.969 (0.510–1.840)	0.923	0.989 (0.520–1.883)	0.974
G (−)	23	0.101	24	0.103	1.032 (0.544–1.960)	0.923	1.011 (0.531–1.924)	0.974
**c.3331 G>A—*IL-6* (rs2069845)**								
*Frequencies*								
G/G	26	0.228	25	0.216	0.930 (0.499–1.733)	0.819	0.917 (0.491–1.712)	0.785
G/A	56	0.491	63	0.543	1.231 (0.733–2.067)	0.431	1.228 (0.730–2.063)	0.439
A/A	32	0.281	28	0.241	0.815 (0.452–1.470)	0.497	0.828 (0.458–1.497)	0.533
χ^2^ = 230.000; *p* = 0.432								
G	108	0.474	113	0.487	1.057 (0.728–1.500)	0.770	1.045 (0.719–1.520)	0.817
A	120	0.526	119	0.513	0.946 (0.652–1.373)	0.770	0.957 (0.658–1.391)	0.817
*Carriage rates*								
G (+)	82	0.360	88	0.379	1.226 (0.680–2.212)	0.497	1.207 (0.668–2.182)	0.533
G (−)	32	0.140	28	0.121	0.815 90.452–1.470)	0.497	0.828 (0.458–1.497)	0.533
A (+)	88	0.386	91	0.392	1.075 (0.577–2.004)	0.819	1.091 (0.584–2.037)	0.785
A (−)	26	0.114	25	0.108	0.930 (0.499–1.733)	0.819	0.917 (0.491–1.712)	0.785
**c.+396 T>G—*IL-8* (rs2227307)**								
*Frequencies*								
T/T	27	0.237	30	0.259	1.124 (0.617–2.047)	0.702	1.120 (0.614–2.042)	0.712
T/G	61	0.535	61	0.526	0.964 (0.574–1.618)	0.889	0.965 (0.574–1.622)	0.894
G/G	26	0.228	25	0.216	0.930 (0.499–1.733)	0.819	0.931 (0.499–1.738)	0.823
χ^2^ = 230.000; *p =* 0.432								
T	115	0.504	121	0.522	1.076 (0.738–1.570)	0.704	1.074 (0.735–1.568)	0.713
G	113	0.496	111	0.478	0.929 (0.637–1.356)	0.704	0.931 (0.638–1.360)	0.713
*Carriage rates*								
T (+)	88	0.386	91	0.392	1.075 (0.577–2.004)	0.819	1.074 (0.575–2.004)	0.823
T (−)	26	0.114	25	0.108	0.930 (0.499–1.733)	0.819	0.931 (0.499–1.738)	0.823
G (+)	87	0.382	86	0.371	0.890 (0.489–1.620)	0.702	0.893 (0.490–1.628)	0.712
G (−)	27	0.118	30	0.129	1.124 (0.617–2.047)	0.702	1.120 (0.614–2.042)	0.712

* Crude OR means OR calculated with conventional logistic regression; adjusted crude OR means OR calculated with conventional logistic regression adjusted for sex.

**Table 3:** Results of two-way ANOVA analyses on mRNA expression of *IL-6* and *IL-8*.

Factor	Gene	Study Groups		Gender/BMI/Smoking		Interaction	
		F	*p*	F	*p*	F	*p* *
Gender	*IL-6*	0.714	0.399	0.717	0.398	0.710	0.400
	*IL-8*	**36.725**	**<0.001**	**11.674**	**<0.001**	**13.151**	**<0.001**
BMI	*IL-6*	1.045	0.308	0.166	0.684	0.162	0.688
	*IL-8*	**24.560**	**<0.001**	0.135	0.713	0.196	0.658
Smoking	*IL-6*	0.932	0.336	0.926	0.337	0.935	0.335
	*IL-8*	**25.526**	**<0.001**	1.059	0.305	0.400	0.528

* *p* < 0.05 are in bold.

**Table 4:** Results of two-way ANOVA analyses on the methylation status of the *IL-6* promoter.

Factor	Study Groups		Gender/BMI/Smoking		Interaction	
	F	*p*	F	*p*	F	*p* *
**Gender**	**10.844**	**0.001**	1.682	0.196	**6.783**	**0.010**
BMI	**13.810**	**<0.001**	0.827	0.364	0.026	0.871
Smoking	**20.848**	**<0.001**	0.681	0.410	0.355	0.552

* *p* < 0.05 are in bold.

**Table 5:** Basic information on the *IL-6* and *IL-8* polymorphic variants.

Gene	SNPs	NCBI db SNP ID	Region	MAF in European Population	Assay ID of TaqMan™ SNP Genotyping Assay	Location	Function	Ref.
*IL-6*	-597 A>G	rs1800797	Intron	G: 0.609	C___1839695_20	Chr.7: 22726602	The G allele was associated with increased inflammatory responses	[22]
	c.3331 G>A	rs2069845	Intron	A: 0.590	C___1839699_10	Chr.7: 22730530	G carriers were characterised by high secretion of IL-6 in serum	[44]
*IL-8*	c.+396 T>G	rs2227307	Intron	G: 0.447	C__11748168_10	Chr.4: 73740952	This polymorphism may regulate the protein production of IL-8	[45]

## Data Availability

The data that support the findings of this study are available on request from the corresponding author [Paulina Wigner; paulina.wigner@biol.uni.lodz.pl].

## Supplementary Materials

The following supporting information can be downloaded at: https://www.mdpi.com/article/10.3390/ijms24076266/s1.
