# Supplementary material for: Variability, Expression, and Methylation of IL-6 and IL-8 Genes in Bladder Cancer Pathophysiology
Source: Int J Mol Sci. 2023 Mar 27;24(7):6266. doi: 10.3390/ijms24076266 (PMC10093978; doi:10.3390/ijms24076266)
Supplement: Supplementary file 1 [file ijms-24-06266-s001.zip › ijms-2300209-supplementary.pdf]

Supplementary materials

Table S1. Gene–gene interactions of *IL-6* and *IL-8* polymorphisms and BC risk.

Combined genotype	Control (n = 114)		BC (n = 116)		Crude OR (95% CI)*	*p*	Adjusted OR (95% CI)*	*p**
	Number	Frequency	Number	Frequency				
**-597 A>G – *IL-6* (rs1800797) and c.+396 T>G – *IL-8* (rs2227307)**								
A/A-T/T	4	0.035	9	0.078	1.615 (0.512-5.093)	0.414	1.662 (0.525-5.267)	0.388
A/A-T/G	11	0.096	11	0.095	1.346 (0.544-3.330)	0.520	1.316 (0.531-3.266)	0.553
A/A-G/G	8	0.070	4	0.034	0.417 (0.125-1.394)	0.155	0.434 (0.129-1.460)	0.178
A/G-T/T	12	0.105	16	0.138	0.732 (0.345-1.553)	0.416	0.715 (0.336-1.521)	0.384
A/G-T/G	33	0.289	33	0.284	0.976 (0.548-1.738)	0.935	0.991 (0.555-1.768)	0.975
A/G-G/G	13	0.114	12	0.103	1.968 (0.800-4.842)	0.141	1.972 (0.800-4.862)	0.140
G/G-T/T	11	0.096	5	0.043	2.037 (0.596-6.964)	0.257	2.062 (0.601-7.074)	0.250
G/G-T/G	17	0.149	17	0.147	0.807 (0.399-1.634)	0.552	0.803 (0.396-1.628)	0.543
G/G-G/G	5	0.044	9	0.078	0.636 (0.219-1.850)	0.406	0.611 (0.209-1.785)	0.368
**c.+396 T>G – *IL-8* (rs2227307) and c.3331 G>A – *IL-6* (rs2069845)**								
T/T-G/G	4	0.035	9	0.078	2.313 (0.691-7.737)	0.173	2.264 (0.675-7.594)	0.186
T/T-G/A	12	0.105	15	0.129	1.262 (0.563-2.830)	0.572	1.227 (0.545-2.759)	0.621
T/T-A/A	11	0.096	6	0.052	0.511 (0.182-1.431)	0.201	0.535 (0.190-1.507)	0.236
T/G-G/G	14	0.123	12	0.103	0.824 (0.364-1.868)	0.643	0.823 (0.362-1.870)	0.642
T/G-G/A	31	0.272	35	0.302	1.157 (0.653-2.050)	0.618	1.171 (0.659-2.079)	0.591
T/G-A/A	16	0.140	14	0.121	0.841 (0.390-1.814)	0.658	0.827 (0.382-1.789)	0.630
G/G-G/G	8	0.070	4	0.034	0.473 (0.138-1.618)	0.233	0.462 (0.135-1.586)	0.220
G/G-G/A	13	0.114	13	0.112	0.981 (0.433-2.218)	0.962	0.980 (0.432-2.222)	0.962
G/G-A/A	5	0.044	8	0.069	1.615 (0.512-5.093)	0.414	1.662 (0.525-5.267)	0.388

Table S2. Synergy factor analysis.

**Genes**	**Polymorphism**	**Subjects**	**Synergy factor[a]**	***p*-value[b]**	**Type interaction[a]**
IL-6 × *IL-8*	-597 A>G (rs1800797) × c.+396 T>G (rs2227307)	G carriers – G carriers	3.259	0.127	Antagonistic
IL-8 × *IL-6*	c.+396 T>G (rs2227307) × c.3331 G>A (rs2069845)	G carriers – A carriers	3.649	0.092	Antagonistic

Table S3. Distribution of haplotypes of the studied polymorphisms of the *IL-6* and risk of the BC.

Haplotype	Control (n = 114)		BC (n = 116)		Crude OR (95% CI)	*p*
	Number	Frequency	Number	Frequency		
**-597 A>G – *IL-6* (rs1800797) and c.3331 G>A – *IL-6* (rs2069845)**						
AG	104	0.456	107	0.461	1.02 (0.707-1.472)	0.913
GA	120	0.526	117	0.504	0.915 (0.635-1.32)	0.636
GG	4	0.017	6	0.025	1.486 (0.413-5.339)	0.540
AA	0	0.000	2	0.008	-	-

Table S4. Correlations between studied SNPs and the clinical characteristics of patients with BC.

**-597 A>G – *IL-6* (rs1800797)**						
Characteristics	Status	Genotype Frequency			χ2	*p*
		AA	AG	GG		
Primary tumour size	Ta	0.389	0.431	0.375	3.346	0.502
	T1	0.389	0.353	0.541		
	≥T2	0.222	0.216	0.125		
Lymph node metastasis	N0	0.778	0.784	0.917	2.146	0.342
	≥N1	0.222	0.216	0.083		
Distant metastasis	M0	0.778	0.784	0.917	2.146	0.342
	M1	0.222	0.216	0.083		
Pathomorphology of nonmuscle-invasive tumors	papillary urothelial neoplasm of low malignant potential (PUN-LMP)	0.250	0.222	0.080	2.967	0.563

Characteristics	Status	Genotype Frequency			χ2	*p*
	low-grade papillary urothelial carcinoma	0.375	0.408	0.440		
	high-grade papillary urothelial carcinoma	0.375	0.370	0.480		

c.3331 G>A – *IL-6* (rs2069845)

Characteristics	Status	Genotype Frequency			χ2	*p*
		GG	GA	AA		
Primary tumour size	Ta	0.368	0.423	0.409	2.128	0.712
	T1	0.421	0.365	0.500		
	≥T2	0.211	0.212	0.091		
Lymph node metastasis	N0	0.789	0.788	0.212	1.629	0.443
	≥N1	0.211	0.909	0.091		
Distant metastasis	M0	0.789	0.788	0.212	1.629	0.443
	M1	0.211	0.909	0.091		
Pathomorphology of nonmuscle-invasive tumors	papillary urothelial neoplasm of low malignant potential (PUN-LMP)	0.240	0.232	0.045	4.261	0.372
	low-grade papillary urothelial carcinoma	0.360	0.411	0.455		
	high-grade papillary urothelial carcinoma	0.400	0.357	0.500		

c.+396 T>G – *IL-8* (rs2227307)

Characteristics	Status	Genotype Frequency			χ2	*p*
		TT	TG	GG		
Primary tumour size	Ta	0.346	0.396	0.526	3.211	0.523
	T1	0.423	0.396	0.421		
	≥T2	0.231	0.208	0.053		
Lymph node metastasis	N0	0.769	0.792	0.947	2.765	0.251
	≥N1	0.231	0.208	0.053		
Distant metastasis	M0	0.769	0.792	0.947	2.765	0.251
	M1	0.231	0.208	0.053		
Pathomorphology of nonmuscle-invasive tumors	papillary urothelial neoplasm of low malignant potential (PUN-LMP)	0.148	0.226	0.174	2.171	0.704
	low-grade papillary urothelial carcinoma	0.519	0.359	0.391		
	high-grade papillary urothelial carcinoma	0.333	0.415	0.435		

Table S5. Distribution of genotypes and alleles of the -597 A>G – *IL-6* (rs1800797), c.3331 G>A – *IL-6* (rs2069845), c.+396 T>G – *IL-8* (rs2227307) and ORs with 95% CIs in men and women with BC.

Genotype/ Allele	WOMEN (n = 71)				MEN (n = 159)			
	Control (n = 39) N (Freq.)	BC (n = 32) N (Freq.)	Crude OR (95% CI)*	*p*	Control (n = 75) N (Freq.)	BC (n = 84) N (Freq.)	Crude OR (95% CI)*	*p*
**-597 A>G – *IL-6* (rs1800797)**								
A/A	7 (0.179)	5 (0.156)	0.847(0.241-2.975)	0.795	16 (0.213)	19 (0.226)	1.078 (0.508-2.288)	0.845
A/G	17 (0.436)	20 (0.625)	2.157 (0.830-5.607)	0.115	41 (0.547)	41 (0.488)	0.791 (0.424-1.476)	0.461
G/G	15 (0.385)	7 (0.219)	0.448 (0.156-1.290)	0.137	18 (0.240)	24 (0.286)	1.267 (0.622-2.578)	0.514
		χ^2 = 70.955; p = 0.380				χ^2 = 158.999; p = 0.418		
A	31 (0.397)	30 (0.469)	1.367 (0.681-2.745)	0.379	73 (0.487)	79 (0.470)	0.934 (0.596-1.463)	0.766
G	47 (0.603)	34 (0.531)	0.732 (0.364-1.469)	0.379	77 (0.513)	89 (0.530)	1.071 (0.684-1.676)	0.766
**c.3331 G>A – *IL-6* (rs2069845)**								
G/G	8 (0.205)	6 (0.188)	0.894 (0.275-2.910)	0.853	18 (0.240)	19 (0.226)	0.926 (0.443-1.933)	0.837
G/A	17 (0.436)	19 (0.594)	1.891 (0.733-4.880)	0.188	39 (0.520)	44 (0.524)	1.015 (0.544-1.894)	0.962
A/A	14 (0.359)	7 (0.219)	0.500 (0.173-1.1448)	0.201	18 (0.240)	21 (0.250)	1.056 (0.512-2.178)	0.884
		χ^2 = 70.971; p = 0.379				χ^2 = 159.000; p = 0.418		
G	33 (0.423)	31 (0.484)	1.290 (0.655-2.539)	0.461	75 (0.500)	82 (0.488)	0.951 (0.606-1.493)	0.828
A	45 (0.577)	33 (0.516)	0.775 (0.294-1.526)	0.461	75 (0.500)	86 (0.512)	1.051 (0.670-1.649)	0.828
**c.+396 T>G – *IL-8* (rs2227307)**								
T/T	9 (0.231)	8 (0.250)	1.111 (0.372-3.315)	0.850	18 (0.240)	22 (0.262)	1.124 (0.547-2.307)	0.751
T/G	21 (0.538)	17 (0.531)	0.971 (0.381-2.480)	0.952	40 (0.533)	44 (0.524)	0.963 (0.516-1.796)	0.904
G/G	9 (0.231)	7 (0.219)	0.933 (0.304-2.864)	0.904	17 (0.227)	18 (0.214)	0.930 (0.439-1.972)	0.851
		χ^2 = 71.000; p = 0.378				χ^2 = 159.000; p = 0.418		
T	39 (0.500)	33 (0.516)	1.017 (0.538-2.125)	0.848	76 (0.507)	88 (0.524)	1.076 (0.683-1.694)	0.753
G	39 (0.500)	31 (0.484)	0.935 (0.471-1.857)	0.848	74 (0.493)	80 (0.476)	0.930 (0.590-1.464)	0.753

Table S6. Distribution of genotypes and alleles of the -597 A>G – *IL-6* (rs1800797), c.3331 G>A – *IL-6* (rs2069845), c.+396 T>G – *IL-8* (rs2227307) and ORs with 95% CIs in BC subjects with normal body weight or subjects with overweight and obesity.

Genotype/ Allele	BMI < 25 (n = 81)				BMI ≤ 25 (n = 149)			
	Control (n = 39)	BC (n = 42)	Crude OR (95% CI)*	*p*	Control (n = 75)	BC (n = 74)	Crude OR (95% CI)*	*p*
	N (Freq.)	N (Freq.)			N (Freq.)	N (Freq.)		
**-597 A>G – *IL-6* (rs1800797)**								
A/A	10 (0.256)	4 (0.095)	0.305 (0.087-1.072)	0.064	13 (0.173)	20 (0.270)	1.766 (0.803-3.884)	0.157
A/G	16 (0.410)	26 (0.619)	2.336 (0.958-5.697)	0.062	42 (0.560)	35 (0.473)	0.705 (0.370-1.344)	0.288
G/G	13 (0.333)	12 (0.286)	0.800 (0.311-2.056)	0.643	20 (0.267)	19 (0.257)	0.950 (0.457-1.973)	0.891
	χ^2 = 81.011; p = 0.385				χ^2 = 149.001; p = 0.416			
A	36 (0.462)	34 (0.405)	0.782 (0.410-1.490)	0.454	68 (0.453)	75 (0.507)	1.250 (0.784-1.990)	0.348
G	42 (0.538)	50 (0.595)	1.279 (0.671-2.438)	0.454	82 (0.547)	73 (0.493)	0.800 (0.502-1.275)	0.348
**c.3331 G>A – *IL-6* (rs2069845)**								
G/G	11 (0.282)	6 (0.143)	0.424 (0.140-1.288)	0.130	15 (0.200)	19 (0.257)	1.382 (0.640-2.983)	0.410
G/A	15 (0.385)	25 (0.595)	2.353 (0.964-5.742)	0.060	41 (0.547)	38 (0.514)	0.875 (0.460-1.666)	0.685
A/A	13 (0.333)	11 (0.262)	0.710 (0.272-1.848)	0.483	19 (0.253)	17 (0.230)	0.879 (0.415-1.863)	0.737
	χ^2 = 81.005; p = 0.386				χ^2 = 149.000; p = 0.416			
G	37 (0.474)	37 (0.440)	0.873 (0.470-1.620)	0.666	71 (0.473)	76 (0.514)	1.187 (0.742-1.900)	0.475
A	41 (0.526)	47 (0.560)	1.146 (0.617-2.127)	0.666	79 (0.527)	72 (0.486)	0.842 (0.526-1.348)	0.475
**c.+396 T>G – *IL-8* (rs2227307)**								
T/T	8 (0.205)	10 (0.238)	1.211 (0.423-3.470)	0.722	19 (0.253)	20 (0.270)	1.092 (0.526-2.267)	0.814
T/G	19 (0.487)	19 (0.452)	0.870 (0.363-2.083)	0.754	42 (0.560)	42 (0.568)	1.031 (0.540-1.971)	0.926
G/G	12 (0.308)	13 (0.310)	1.009 (0.393-2.591)	0.986	14 (0.187)	12 (0.162)	0.843 (0.361-1.970)	0.694
	χ^2 = 81.000; p = 0.386				χ^2 = 149.000; p = 0.416			
T	35 (0.449)	39 (0.464)	1.061 (0.581-1.939)	0.847	80 (0.533)	82 (0.554)	1.102 (0.674-1.800)	0.699
G	43 (0.551)	45 (0.536)	0.942 (0.516-1.722)	0.847	70 (0.467)	66 (0.446)	0.908 (0.556-1.483)	0.699

Table S7. Distribution of genotypes and alleles of the -597 A>G – *IL-6* (rs1800797), c.3331 G>A – *IL-6* (rs2069845), c.+396 T>G – *IL-8* (rs2227307) and ORs with 95% CIs in non-smokers and smokers with BC.

Genotype/ Allele	NON-SMOKER (n = 104) Control (n = 68) N (Freq.)	BC (n = 36) N (Freq.)	Crude OR (95% CI)*	p	SMOKER (n = 126) Control (n = 46) N (Freq.)	BC (n = 80) N (Freq.)	Crude OR (95% CI)*	p
**-597 A>G – *IL-6* (rs1800797)**								
A/A	15 (0.221)	11 (0.306)	1.555 (0.625-3.869)	0.343	8 (0.174)	13 (0.163)	0.922 (0.351-2.423)	0.869
A/G	32 (0.471)	15 (0.417)	0.804 (0.355-1.817)	0.599	26 (0.565)	46 (0.575)	1.041 (0.500-2.165)	0.915
G/G	21 (0.309)	10 (0.278)	0.861 (0.353-2.101)	0.742	12 (0.261)	21 (0.263)	1.008 (0.442-2.302)	0.984
		χ^2 = 104.041; p = 0.398				χ^2 = 126.000; p = 0.408		
A	62 (0.456)	37 (0.514)	1.237 (0.715-2.143)	0.447	42 (0.457)	72 (0.450)	0.969 (0.554-1.697)	0.913
G	74 (0.544)	35 (0.486)	0.808 (0.467-1.399)	0.477	50 (0.543)	88 (0.550)	1.032 (0.589-1.806)	0.913
**c.3331 G>A – *IL-6* (rs2069845)**								
G/G	17 (0.250)	12 (0.333)	1.500 (0.620-3.631)	0.369	9 (0.196)	13 (0.163)	0.798 (0.312-2.042)	0.637
G/A	31 (0.456)	17 (0.472)	1.068 (0.475-2.400)	0.874	25 (0.543)	46 (0.575)	1.136 (0.548-2.359)	0.731
A/A	20 (0.294)	7 (0.194)	0.579 (0.218-1.538)	0.273	12 (0.261)	21 (0.263)	1.008 (0.442-2.302)	0.984
		χ^2 = 103.953; p = 0.400				χ^2 = 126.004; p = 0.408		
G	65 (0.478)	41 (0.569)	1.410 (0.806-2.468)	0.228	43 (0.467)	72 (0.450)	0.922 (0.530-1.604)	0.774
A	71 (0.522)	31 (0.431)	0.709 (0.405-1.241)	0.228	49 (0.533)	88 (0.550)	1.084 (0.623-1.887)	0.774
**c.+396 T>G – *IL-8* (rs2227307)**								
T/T	14 (0.206)	10 (0.278)	1.484 (0.581-3.785)	0.409	13 (0.283)	20 (0.250)	0.846 (0.374-1.916)	0.689
T/G	40 (0.588)	19 (0.528)	0.782 (0.347-1.764)	0.554	21 (0.457)	42 (0.525)	1.316 (0.636-2.723)	0.460
G/G	14 (0.206)	7 (0.194)	0.931 (0.338-2.565)	0.890	12 (0.261)	18 (0.225)	0.823 (0.355-1.909)	0.649
		χ^2 = 104.034; p = 0.398				χ^2 = 126.000; p = 0.408		
T	68 (0.500)	39 (0.542)	1.214 (0.654-2.251)	0.539	47 (0.511)	82 (0.513)	1.007 (0.603-1.682)	0.980
G	68 (0.500)	33 (0.458)	0.824 (0.444-1.528)	0.539	45 (0.489)	78 (0.488)	0.993 (0.595-1.660)	0.980

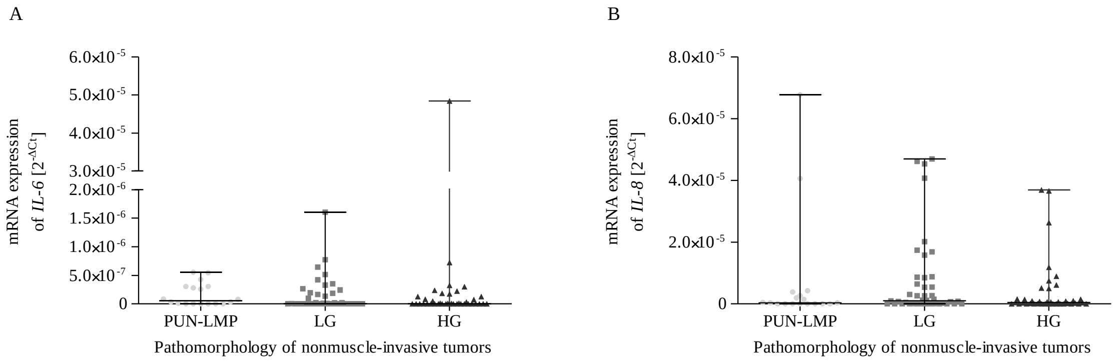

Figure S1. Basic *IL-6* (**A**) and *IL-8* (**B**) expression in the subgroups of the papillary urothelial neoplasm of low malignant potential (PUN-LMP), low-grade papillary urothelial carcinoma (LG), high-grade papillary urothelial carcinoma (HG) , expressed as $2^{-\Delta Ct}$ ($C_{t\ gene} - C_{t\ 18S}$) method for each sample. The data are plotted as individual values and the median with interquartile range is indicated by the horizontal bars.

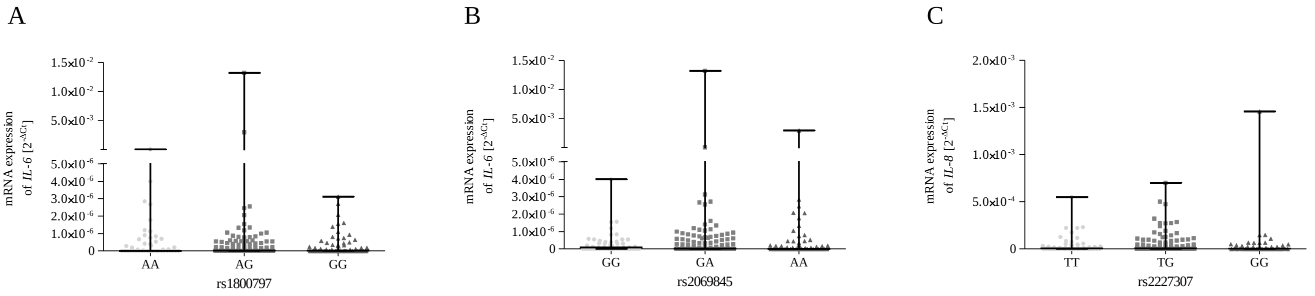

Figure S2. Distribution of single nucleotide polymorphisms of genes encoding *IL-6* (**A**, **B**) and *IL-8* (**C**) and mRNA expression level of *IL-6* and *IL-8* expressed as $2^{-\Delta Ct}$ ($C_{t\ gene} - C_{t\ 18S}$) method for each sample. The data are plotted as individual values and the median with interquartile range is indicated by the horizontal bars.

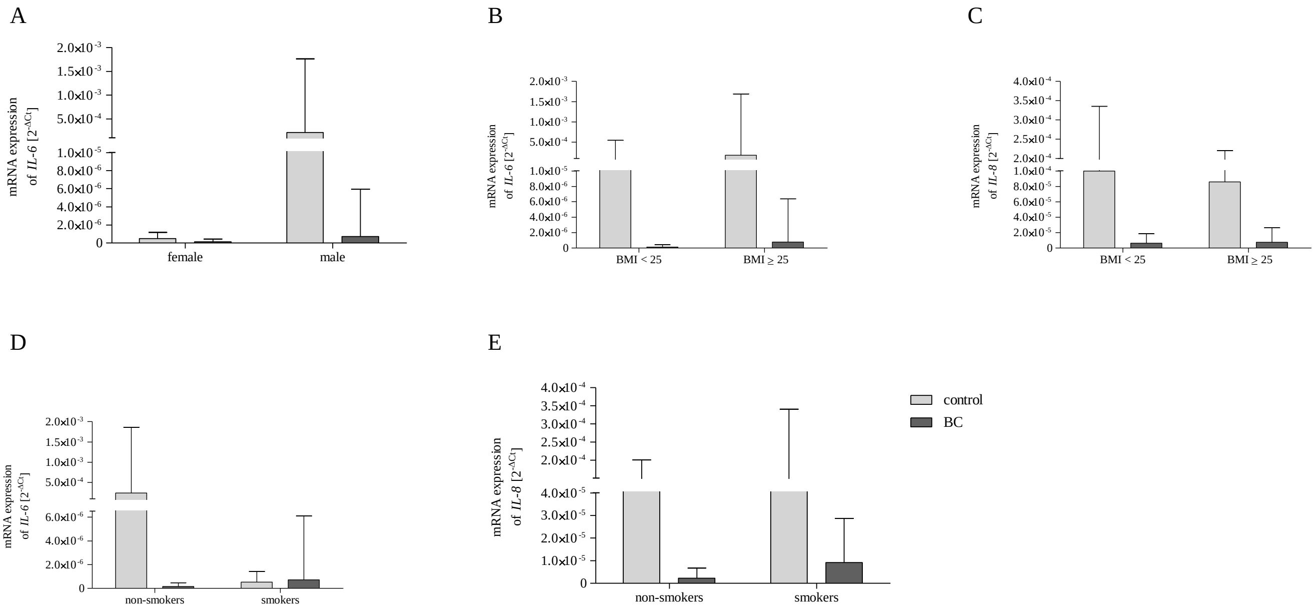

Figure S3. Two-way ANOVA with Bonferroni post hoc test show significant effects of BC and sex on *IL-6* expression (**A**), BMI on the mRNA expression of *IL-6* (**B**) and *IL-8* (**C**) as well as cigarette smoking on *IL-6* (**D**) and *IL-8* (**E**) expression. Gene expression in PBMCs has expressed as $2^{-\Delta Ct}$ ($C_{t\ gene} - C_{t\ 18S}$) method. The data are presented as mean ± SD.

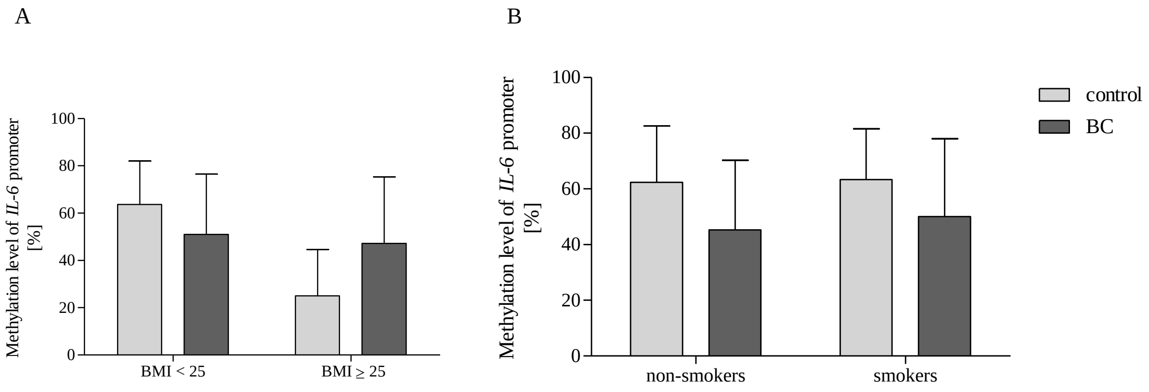

Figure S4. Two-way ANOVA with Bonferroni post hoc test show significant effects of BC and BMI (**A**) or cigarette smoking (**B**) on the methylation status of *IL-6* promoter region The data are presented as mean ± SD.
